# Needle Found in the Heart of a Cow

**Published:** 1846-01-01

**Authors:** J. H. Beech

**Affiliations:** Gaines, N. Y.


### Needle found in the Heart of a Cow.

Communicated for the Buffalo Medical Journal, by J. H. BEECH, M. D., of Gaines, N. Y.

Dr. Flint: Sir—having recently become somewhat acquainted with you, through your excellent journal, I take the liberty of sending to you the following circumstances, thinking that the case may be rather interesting to yourself, if it be not worthy of any further notice; of this you must judge, and please use it as you think proper.

About four years ago, Mr. Jas. Mather, of this village, requested me to examine the body of a cow which had just died, being in very good flesh. He had owned the cow about two years: she had been sick at short intervals during most of the time, and recently had appeared to be distressed for breath. I found in the pericardium two or three quarts of thinnish, purulent, *acrid* matter. In taking out the heart, my finger was pricked by what I found to be the point of a large darning needle. I think its track could be seen from the œsophagus; it seemed to have entered the right ventricle just below the middle, had passed directly through, and was fixed across the left ventricle about through the middle, with the point sticking out on the left side 1-4 of an inch. There was slight hypertrophy of the walls of the ventricles; otherwise the organ appeared healthy. Some congestion existed in a small portion of the left lung. These were all the signs of disease which I saw; the weather was very cold, and I was unable to make as close an examination as I would have liked.

I have the needle now in my possession; it is very much rusted, but the eye is still entire.

This cow had been fed on "*slops*" by a former owner, but not while in Mr. M.'s possession. I think she must have got the needle in that way, and that it had been in the body for more than two years, and for a long time in the substance of the heart.

Very respectfully your ob't serv't J. H. BEECH.

*Gaines, November* 23, 1845.

